# Goal setting for WHO guideline adherence: accelerometer steps/day translation of moderate-to-vigorous physical activity in older adults

**DOI:** 10.3389/fpubh.2025.1575209

**Published:** 2025-07-30

**Authors:** I-Fan Chen, Jiaren Chen, Ting-Fu Lai, Yung Liao, Jong-Hwan Park, Chih-Fu Cheng

**Affiliations:** ^1^Country Department of Physical Education and Sports Science, National Taiwan Normal University, Taipei, Taiwan; ^2^Graduate Institute of Sport, Leisure and Hospitality Management, National Taiwan Normal University, Taipei, Taiwan; ^3^Biomedical Research Institute, Pusan National University Hospital, Busan, Republic of Korea; ^4^Faculty of Sport Sciences, Waseda University, Tokorozawa, Japan; ^5^Department of Convergence Medicine, Pusan National University School of Medicine, Yangsan, Republic of Korea; ^6^Department of Clinical Bio-Convergence, Graduate School of Convergence in Biomedical Science, Pusan National University School of Medicine, Yangsan, Republic of Korea; ^7^Convergence Medical Institute of Technology, Pusan National University Hospital, Busan, Republic of Korea

**Keywords:** daily steps, WHO guideline, walking, accelerometer, physical activity, older adults

## Abstract

**Background:**

Walking is a fundamental and accessible physical activity (PA) for older adults. Using daily step counts to self-monitor adherence to the World Health Organization’s (WHO) PA guidelines may facilitate health promotion and promote behavioral changes. This study aimed to determine the step count equivalents for achieving 30 min/day and 150 min/week of moderate-to-vigorous PA (MVPA) among older adults in Taiwan.

**Methods:**

This cross-sectional study was conducted from May to August 2023. Community-dwelling adults aged ≥65 years were recruited through convenience sampling from local communities in northern Taiwan, with support from community leaders and activity centers. Participants were required to be capable of walking independently. Daily step counts and MVPA were measured using triaxial accelerometers. Linear regression models, including log-transformed step counts and MVPA, were used to assess equivalence.

**Results:**

In total, 191 older adults participated in the study (42 males, 149 females). Engaging in 30 min of MVPA daily translated to approximately 8,602 steps/day for males and 8,940 steps/day for females. Weekly MVPA of 150 min was equivalent to approximately 56,028 steps/week for males, 58,988 steps/week for females, and 57,000 steps/week overall (or 8,142 steps/day).

**Conclusion:**

Among older adults, reaching at least 8,602 steps/day is a good indicator of meeting the WHO’s recommendation of 30 min/day MVPA, whereas 57,000 steps/week corresponds to 150 min/week of MVPA. These step-based thresholds provide practical and easy-to-understand targets for self-monitoring daily activity levels and offer a behavioral foundation for PA promotion strategies aimed at improving the health of aging populations.

## 1 Introduction

Walking plays a central role in supporting physical independence, mobility, and overall health in older adults. For many community-dwelling older adults with intact mobility, walking is often a relatively accessible form of physical activity (PA) that does not typically require specialized equipment or environments, and can be performed in a variety of everyday settings. Furthermore, previous evidence has consistently demonstrated the health benefits of walking among older adults, including decreased risks of obesity (1), cardiovascular disease (2), and mortality (3, 4). Walking has also been shown to be an effective way to reduce anxiety and alleviate symptoms of depression (5). Moreover, higher step counts have notably been associated with lower national healthcare costs (6, 7). This makes walking an ideal activity for promoting behavioral changes and encouraging healthier lifestyles among older adults.

According to the 2020 World Health Organization (WHO) guidelines on PA and sedentary behavior, older adults should engage in at least 150 min of moderate-intensity PA (MPA) or 75 min of vigorous-intensity PA (VPA) per week or an equivalent combination of MPA and VPA per week (moderate-to-vigorous physical activity [MVPA]) (8). However, achieving MVPA can be challenging for older adults or those with mobility limitations or chronic conditions, whereas light-intensity PA (LPA), such as walking, may be more feasible (9). Despite these recommendations, older adults may struggle to assess whether they meet the WHO guidelines in their daily lives. Establishing a step-based equivalent for MVPA could provide a practical and objective target for self-monitoring in older populations. Furthermore, only three studies available examined the step-count equivalent of MVPA. Two of these studies focused on adults aged 20 years and older from European populations, with one study finding that 30 min of MPA per day is equivalent to 3,000 steps (10), while another study found that the same duration of MVPA is equivalent to approximately 8,000 steps (11), with differences between males and females. The third study, which focused on children and adolescents from European populations, found that 60 min of MVPA per day was equivalent to 9,000 steps (12). These studies primarily involved younger adults, and there is a lack of research specifically involving older adults, particularly Asian populations.

Previous studies have used various methods to assess mobility and PA in older adults, including subjective self-report questionnaires—most commonly the International Physical Activity Questionnaire (IPAQ)—as well as objective tools such as accelerometers and pedometers. Compared to self-reported measures, which may be affected by recall bias (13), accelerometers offer higher validity and can capture not only step counts but also the intensity of PA, making them a more robust tool for measuring free-living activity in older adults.

Thus, this study aimed to determine the step count equivalent to daily MVPA among older adults in Taiwan using triaxial accelerometers and, based on the WHO guidelines on PA and sedentary behavior, to establish a weekly step count equivalent to 150 min of MVPA. Based on goal-setting theory, these findings are expected to provide a scientific basis for self-monitoring behavior among Taiwanese older adults and serve as empirical evidence for future health promotion policies aimed at enhancing PA, encouraging positive behavioral changes, and promoting healthy aging in older populations.

## 2 Materials and methods

### 2.1 Participants and data collection

From May to August 2023, community-dwelling older adults aged 65 years and older in northern Taiwan were recruited through convenience sampling from local community settings. Participants were invited to join the study through community leaders and promotional activities held at community activity centers. For the current study, which focused on accelerometer data, eligible participants were required to be capable of walking independently and willing to wear a triaxial accelerometer around their waist for seven consecutive days. The research staff thoroughly explained the study procedures to each potential participant, including the purpose of the accelerometer data collection as well as the overall study design. Written informed consent, consistent with the principles outlined in the Declaration of Helsinki, was obtained from each participant before enrollment. Following the provision of informed consent, participants completed a sociodemographic questionnaire that collected basic information such as age and sex. They were then fitted with an accelerometer and instructed on its proper use. Upon completion of the data collection period, the participants received a $7 voucher as a token of appreciation for their time and contribution to the study.

### 2.2 Measurements

Daily step counts and MVPA were quantified using a triaxial accelerometer (Ac-ti-Graph GT3X+, Pensacola, FL, USA), which is known for its reliability in monitoring older populations (14). The device was worn on the waist, as previous studies have shown that wrist placement may lead to overestimation of PA due to excessive arm movements. In contrast, waist placement has been demonstrated to provide more accurate measurements of walking-related activity (15). Participants were instructed to wear the device continuously over a seven-day period, except during water activities such as swimming or bathing. Sleep times were also recorded by the participants and treated as non-wear time in the analysis. Valid accelerometer data were defined as (i) ≥ 600 min of a day and (ii) at least four valid days (consisting of three weekdays and one weekend day), which are widely used criteria in previous accelerometer-based studies to ensure reliable estimates of habitual activity (16). Non-wear time was de-fined as any 60-min period during which the device recorded zero counts, including reported sleep periods. Given that the participants in this study were community-dwelling older adults, we defined time spent on MVPA as minutes accumulated at ≥2020 counts/min (17), a threshold that aligns with a previous nationwide study and has been widely adopted in studies involving older adults (11). However, Copeland and Esliger (18) proposed a lower threshold of ≥1,040 counts/min for older adults, which may include light-intensity PA and thus overestimate MVPA. Data processing was performed using ActiLife software (version 6.0, Pensacola, FL, USA) configured to a 60-s epoch and a sampling rate of 30 Hz.

### 2.3 Statistics analyses

The MVPA minutes were averaged across valid days to yield daily estimates, where-as the accumulated daily steps were averaged to provide daily step counts. Initially, logarithmic transformations of the average daily steps and MVPA were correct for non-normally distributed data (19). This is a standard approach in statistical analysis to improve normality and stabilize variance when dealing with positively skewed variables (20). Using a linear regression model, the natural logarithm of daily steps served as an independent variable against the natural logarithm of daily minutes spent on MVPA, which was the dependent variable. Furthermore, in line with the WHO’s guidelines on physical activity and sedentary behavior for older populations, which recommend at least 150 min of MVPA per week, we used a regression model to analyze the predicted steps/day (summed over the week), corresponding to a minimum of 150 min/week of MVPA. This approach helped elucidate the relationship between average daily step counts and their transformation into weekly MVPA durations.

## 3 Results

Table 1 presents the characteristics of the participants. In total, 191 participants (42 males and 149 females) were included in this study. The mean participant age was 71.9 ± 5.8 years, with males being slightly older than females (73.6 ± 5.5 vs. 71.4 ± 5.7 years). Participants accumulated an average of 21.5 ± 18.9 min of MVPA per day, with males aver-aging 21.4 ± 19.5 min/day and females 21.6 ± 18.7 min/day. The overall mean daily step count was 7300.5 ± 3150.3 steps/day, with males averaging 6968.1 ± 3290.9 steps/day and females 7392.8 ± 3116.4 steps/day.

**Table 1:** Characteristics of participants (*n* = 191).

Variables	Overall (*n* = 191) Mean ± SD	Male (*n* = 42) Mean ± SD	Female (*n* = 149) Mean ± SD
Age (years)	71.9 ± 5.8	73.6 ± 5.5	71.4 ± 5.7
MVPA (min/day)	21.5 ± 18.9	21.4 ± 19.5	21.6 ± 18.7
Step Count (steps/day)	7300.5 ± 3150.3	6968.1 ± 3290.9	7392.8 ± 3116.4

SD: standard deviation; MVPA: moderate-to-vigorous physical activity.

Table 2 provides the estimated daily and weekly step counts corresponding to 30 min of MVPA per day and 150 min of MVPA per week, categorized by sex. By applying antilogarithms to revert the predictive model to its original scale, the equations used to estimate daily steps based on MVPA time were as follows: for males, Y = 2544.6 + 1781.6 × LnMVPA (R^2^ = 0.53, *p* < 0.001); and for females, Y = 2691.5 + 1837.8 × LnMVPA (R^2^ = 0.55, *p* < 0.001). Therefore, participants engaging in 30 min of MVPA per day were estimated to have achieved approximately 8,602 steps/day for males or 8,940 steps/day for females.

**Table 2:** Estimated daily and weekly step counts corresponding to 30 min/day and 150 min/week of moderate-to-vigorous physical activity in older adults by sex.

MVPA Equivalent	Male (steps)	Female (steps)	Overall Estimate (steps)
30 min/day	8,602 steps/day	8,940 steps/day	—
150 min/week	56,028 steps/week	58,988 steps/week	57,000 steps/week (or 8,142 steps/day)

MVPA: moderate-to-vigorous physical activity.

For weekly steps associated with 150 min/week of MVPA, based on a subsample of participants with seven valid days of data, the sex-specific prediction equations are Y = −6215.6 + 12423.8 × LnMVPA for males (R^2^ = 0.53, *p* < 0.001) and Y = −18057.5 + 15378.3 × LnMVPA for females (R^2^ = 0.62, *p* < 0.001). These equations correspond to approximately 56,028 steps/week for males and 58,988 steps/week for females, or an average of 57,000 steps/week (equivalent to 8,142 steps/day) as a suitable general estimate.

**Figure 1:**
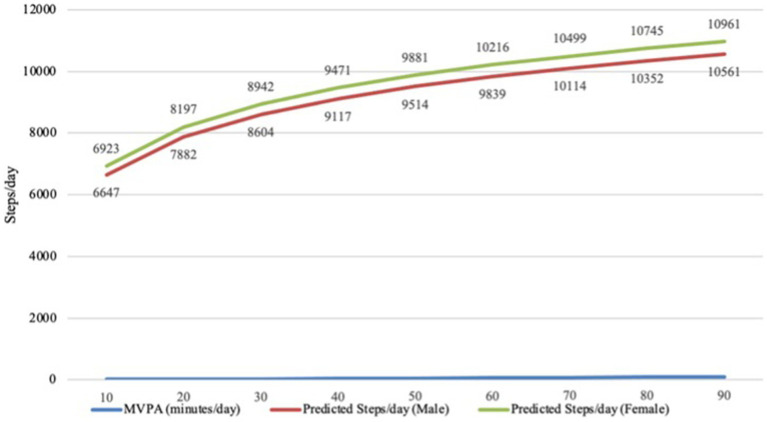
Correlation between daily step counts and moderate-to-vigorous physical activity in older adults by sex.

Figure 1 illustrates the translation of MVPA, accumulated in 10-min daily increments, into the corresponding daily step counts for both males and females. Predicted daily step counts are presented for both sexes based on incremental increases in daily MVPA.

## 4 Discussion

This study is the first to examine the step-count equivalent of MVPA among older adults in Taiwan. The findings indicate that accumulating approximately 8,602 steps/day for males and 8,940 steps/day for females is equivalent to engaging in 30 min of MVPA per day. Additionally, achieving 56,028 steps/week for males, 58,988 steps/week for females, or 57,000 steps/week overall (or 8,142 steps/day) aligns with the WHO’s recommendation of 150 min of MVPA per week.

These results are consistent with those of previous research in the United States (11), which found that males required 7,900 steps/day and females 8,300 steps/day to meet the same MVPA threshold. Similar to the findings of that study, our results indicate that females require more steps than males, with an overall daily recommendation of approximately 8,000 steps. One possible explanation for this difference is that older females may engage in more household activities, leading to higher step counts (21). However, the step counts observed in our study were slightly higher than those reported previously. One possible explanation for this finding is ethnic differences. Cultural differences in daily routines may also contribute, as older adults in Taiwan may prefer walking for transportation or daily errands. Another explanation is that older adults may accumulate more steps because of their greater leisure time than younger adults, leading to more opportunities for LPA, such as walking (22). Moreover, the R^2^ values (0.53 for males and 0.55 for females) indicate moderate model fit, suggesting that factors beyond MVPA—such as environmental conditions or functional status—may affect the accuracy of step count estimation (23). Future studies should consider incorporating such variables to enhance model precision. Based on this evidence, a daily step goal of at least 8,600 steps is recommended for older adults to meet the WHO guidelines and support overall health. Similarly, achieving 57,000 steps/week (or 8,142 steps/day) aligns with the WHO’s 150-min weekly MVPA guidelines. This is comparable to findings from adult studies in the United States (11), which identified 49,000 steps/week as the equivalent threshold. As previously noted, the higher step count observed in this study may be attributed to the increased leisure time among older adults, resulting in greater engagement in daily walking.

Given that walking is the most accessible form of PA for older adults, requires no specialized equipment, and is feasible regardless of physical condition, the step-count targets presented in this study can serve as a practical reference for the self-monitoring of PA behavior. The widespread availability of smartphones and other commercial wearable devices further enhances the feasibility of meeting these targets in older populations. These devices provide convenient and accurate step-tracking, making it easier for individuals to stay motivated, track their progress, and ultimately improve their long-term health outcomes (24). These findings may inform the development of step-based goals in public health campaigns and be integrated into wearable device algorithms tailored for older adults. However, as this study focused on community-dwelling older adults with independent mobility, the applicability of the findings to those with mobility limitations or chronic conditions should be interpreted with caution. Future research is warranted to explore step-based recommendations for more specific subgroups of older adults to provide more tailored and practical guidance.

A major strength of this study was the use of objective measurement tools to enhance the reliability of the findings. However, this study had several limitations. First, the participants were recruited through convenience sampling in Northern Taiwan, where older adults may be more physically active. This may limit the generalizability of the findings to the broader older adult population in Taiwan. Second, the sample had an uneven sex distribution, which may have influenced the observed step count differences. Although sex-specific step-count thresholds were estimated separately, the predominance of female participants may have resulted in overall estimates that more closely reflect activity patterns typical of older women. Future research should include a larger and more diverse cohort to improve the generalizability and robustness of these findings. Third, this study did not include direct measures of health outcomes, as the primary aim was to establish step-count equivalents of MVPA for use in daily self-monitoring among older adults. Overall, it is noted that this study may be subject to selection bias in sampling and confounding bias. Future research could incorporate health-related indicators to validate whether the recommended step thresholds are associated with meaningful improvements in physical or mental health.

## 5 Conclusion

This study recommends that older males and females should target at least 8,602 and 8,940 steps per day, respectively. Overall, this study indicates that accumulating at least 57,000 steps per week (or 8,142 steps per day) is a reasonable target for older Taiwanese adults to meet the WHO recommendation of 150 min of PA per week. Furthermore, these findings provide evidence-based daily step count guidelines for promoting PA in this population and offer a practical reference for self-monitoring in daily life. Future studies should validate these findings using larger sample sizes to strengthen the evidence base.

## Data Availability

The datasets generated and/or analyzed during the current study are not publicly available due to participant privacy and confidentiality restrictions. Requests to access the datasets should be directed to parkj@pusan.ac.kr.
